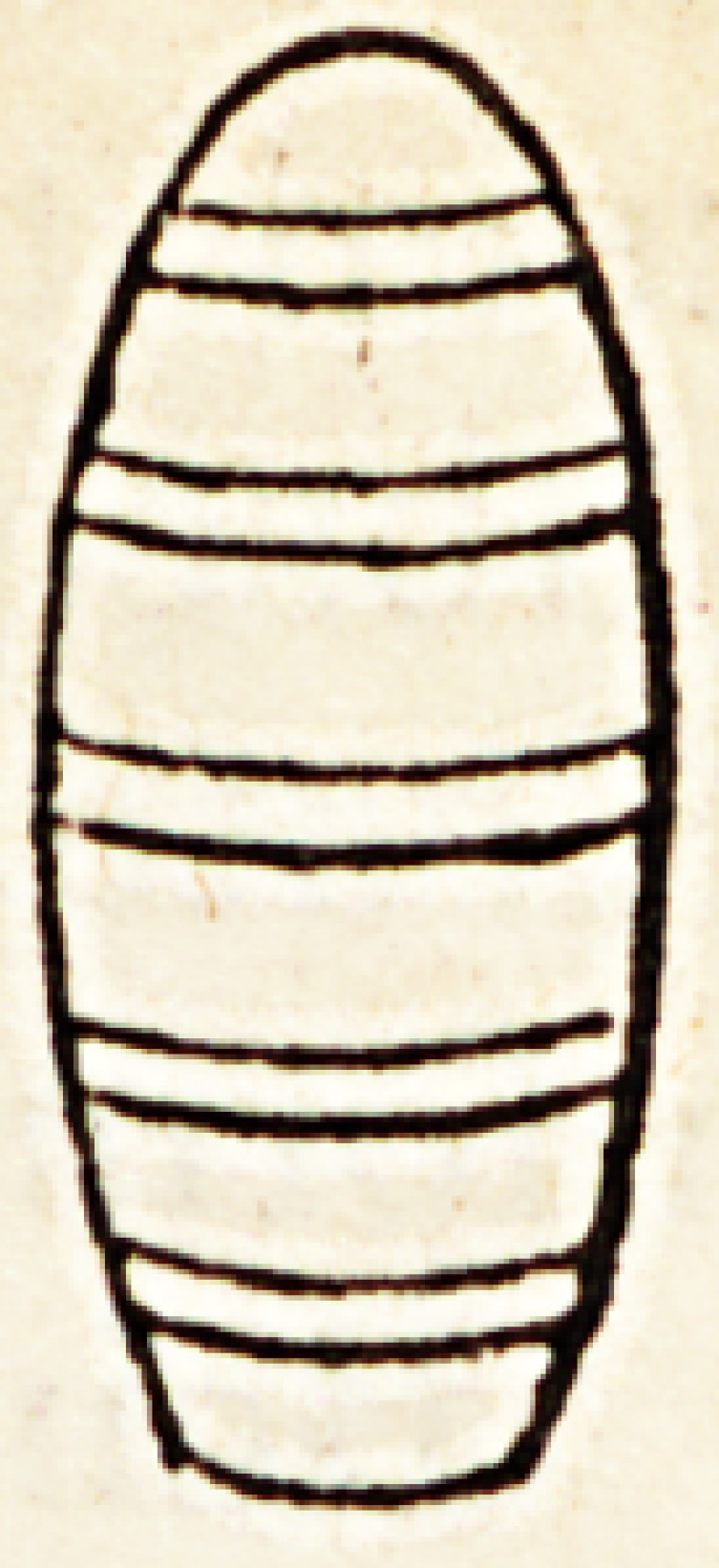# Some Account of Two Cases of Inflammatory Tumour, Produced by the Deposit of the Larva of a Large Fly (Œstrus Humanus) beneath the Cutis, in the Human Subject: Accompanied with *Drawings* of the Larva

**Published:** 1834-10-01

**Authors:** John Howship


Mr. Howship's Cases of Œstrus Humanus.

London. J. Souter. 73, St. Pauls Church Yard.

*Some Account of two Cases of Inflammatory Tumour, produced by the Deposit of the Larva of a large Fly (Œstrus Humanus) beneath the Cutis, in the Human Subject; accompanied with Drawings of the Larva.* By JOHN HOWSHIP, Esq.

(See the Plate.)

THE stomach and alimentary canal of animals are known to afford support to several species of worms, and occasionally also to various insects: indeed, all the cavities communicating with the intestinal canal, and even the urinary passages, are subject to the occasional intrusion of insects, some of which are ascertained, and others not yet determined.

Some years since, in conversation, an intelligent friend mentioned having once seen, in Surinam, the larva of some large insect lodged in an inflammatory tumour beneath the cutis, in one of the men under his charge. His statement of the fact was too clear to admit of doubt, although to me it was so entirely new, that it made a deep impression; the only animals perhaps yet known to take up their abode in the subcutaneous cellular tissue of the human species being the Dracunculus, or guinea-worm, and the Furia Infernalis; the Pulex Penetrans, or Chigoe, forming its nidus beneath the cuticle only.*

In August, 1832, I had the pleasure of seeing a young gentleman, formerly one of my house-pupils, on his return to England. He had obtained the appointment of surgeon to a mining company, at a settlement near Santa Anna, Colombia, where he had been resident several years. On this occasion I was agreeably surprised by his presenting me with a specimen of a larva, which he had himself pressed out from a tumour. The case, he said, he had preserved for me; but as it was, with his baggage, not yet arrived, I noted the particulars at the time from his verbal statement.

It now appeared practicable to ascertain with some precision the particular description of fly to which the larva in question might be referred; but, on shewing it to Mr. Clift, he said he recollected having once, and only once, seen a similar specimen, with a very similar history, at the sale of the collection of the late Mr. T. Keate, surgeon-general to the army, although he did not know what became of it. It may be presumed that Mr. Keate had obtained his specimen

* For a knowledge of the existence of the Furia I am indebted to my distinguished friend, Mr. Hatchett, who, when I had the pleasure of reading these remarks to him, and mentioning the two other insects, immediately gave me the name of the third; referring me to the system of Linnæus, where it has a place, and also to Coxe's Travels in Russia, in which the severe, and even fatal effects, produced by its lodgment beneath the skin are well described.

as a curiosity presented by some gentleman, who, having seen the case, had brought the insect home with him. I subsequently mentioned the circumstance to Mr. Robert Keate, who regretted it was not in his power to give me any information regarding it.

Being told that some such instance had been placed on record, I consulted, but without success, several works to which I was referred; although I still doubt whether such notice may not somewhere be found.*

Under these circumstances the best course appeared to be, to obtain from good authority a description of the larva, including its probable place in a systematic view; together with those suggestions that might be useful in any future case, by enabling the observer to preserve the larva alive, until, breaking from its confinement in the pupa, it came forth, the perfect fly: thus affording the most desirable result, and only adequate means for determining its exact place in the extensive arrangement of natural history; and that it would then be desirable so to make known these observations as to meet the eye and obtain the attention of gentlemen who, from their professional duties abroad, may have the opportunity of following up, and completing, the present inquiry.

With these objects in view, I obtained, through my friend, Mr. Houlton, surgeon, an introduction to Mr. J. Curtis, an active member of the Linnæan Society; a gentleman, the extent of whose scientific acquirements is only equalled by the zeal with which he devotes his time and talents to the study of natural history, and most especially entomology. To this gentleman I am indebted for his valuable descriptive remarks subjoined, and for his attention in procuring the annexed drawings of the insect, by an artist of singular merit, exclusively devoted to these pursuits.

In reference to the first of the above-mentioned cases, I lately wrote to my friend, Mr. W. Gill, many years a surgeon in the army, and now a teacher of anatomy in Liverpool, who favoured me, in reply, with the following statement:

"In August, 1806, I visited a soldier of the 64th regiment, at an outpost, on the Marawina river, Surinam, with a boil, of the size of half-a-crown, on his back, a little below the scapula: it was acuminated, and sloping towards the margin.

* Having recently heard that this insect is described in Humboldt's Travels in South America, to ascertain the fact, I referred to the original copy, in the library of the British Museum. I read through the references to the plates on natural history, looked over the plates themselves, and also the letterpress bearing upon the plates, without finding it; yet it is possible I might have overlooked it.

Compression ejected a maggot, or larva, about this size and form. Horizontal, dirty brownish stripes were on both surfaces, (I believe I am right,) which were alike somewhat convex. Between the stripes the colour was of a dirty white. Unfortunately it was not preserved, nor indeed accurately examined. I think the man was aware he had, as he said, a worm in his boil. The outpost (Prince William Frederick) was on a sandbank, closely hemmed in by the bush (wood.) The water for drinking was procured by sinking two rum puncheons into the sand, close to the sea, without bottom or head.

"The barracks were occasionally visited by the Vespertilio Cynocephalus, which sometimes bled us. One night, for example, I went to sleep without my mosquito-net: I awoke, and, placing my hand on my arm, found a small quantity of blood, which had flowed from a circular bite in the skin, effected by one of these gentlemen. It healed readily. Of course, in such a country, and such a position, we ate, drank, and respired animalculæ in abundance. The back water, in the woods, swarmed with fish; in the river, we had the Manatus. The wind blowing on us from the sea, it was healthy in the extreme. No ailment visited us, save an occasional dysenteric attack."

The following are the notes I took regarding the second case.

August 29th, 1832. Mr. G. Treherne brought me the larva of an insect, which he took from beneath the cutis of a patient, while on duty at Santa Anna, in the district of Maraquita, Colombia.

A young man, a carpenter, requested him to look at his scrotum, upon the anterior inferior part of which was an inflamed swelling, more than an inch in diameter, with two small ulcerated openings, discharging a thin purulent matter, little painful, but rather itching: he sometimes, however, complained of a smarting pain. It was intended, if the two little openings did not heal, to lay them into one.

In a day or two he saw him again, (the swelling having existed several months,) and then observed something white occupying the largest of the openings. Mr. Treherne, gently pressing the tumour, perceived a whitish substance advance and recede. He at length suspected it must be an insect, and, continuing to press it, the larva was further protruded, as the opening relaxed; and, with the effusion of a drop or two of blood, the thickest part soon escaped, when it dropped out, and fell to the ground. It was lively, turned to and fro, protruded its extremities, and retracted them again.

On the escape of the larva, the little cavity very soon healed.

It is worthy of remark, that both these cases occurred in nearly the same parallel of latitude, Surinam and Santa Anna being each situated about five or six degrees north.

It now only remains, in conclusion, to add the following valuable statement, with the description of the larva, by Mr. Curtis.

"The insects that compose this remarkable group of flies have been divided into three genera, principally distinguished by the neuration of their wings; and it is interesting to find they also vary considerably in their economy. Two of these genera are inhabitants of Europe, and the third is confined, I believe, to North America. The former are named Œstrus,* and Gasterophilus;† and by the English they are denominated Bots and Gadflies. The first of these genera, containing several species, live, in their larva state, under the skin of animals, in the fauces, near the root of the tongue, or the frontal sinuses of the head; the latter, containing four described species, inhabit the stomach of the horse; and one of the American group, called, by Mr. Bracy Clark, *Cuterebra*,‡ resides under the skin of the rabbit.

From the economy of the larva submitted to my inspection, and considering the country it came from, it might fairly be inferred that it was related to Cuterebra; but, on a careful examination, it appears to me to resemble most strongly some that have been transmitted to me, that were taken from the fauces of deer, the fly from which I believe to be the Œstrus Pectus of the British Entomology. It is spined in a similar way, and appears to have two hooks over the mouth, which are said, by M. Reaumur and Mr. Clark, to be wanting in the larvæ of the Œstrus Bovis.

Without, therefore, having a specimen of the fly, it is impossible to say to which of these genera the insect belongs; perhaps to none hitherto described.

For the sake of identifying the insect in future investigations, I shall propose calling it for the present *Œstrus hu-*

* Curtis's British Entomology, vol. iii., fol. and plate 106.
† Ibidem, - - - - - 146.
‡ Clark's Essay on the Bots of Horses and other Animals.

*manus*;* and it may be thus characterised: order *Diptera*, family *Muscidæ*, genus *Œstrus?*

*Œstrus humanus*, Curt., fig. 1. The underside, fig. 2, side view. *a, a*. The head. Larva more than an inch long, subfusiform-ovate, dull ochrous colour, most attenuated and brown towards the tail, wrinkled, composed of nine rings. Besides the head, the first six, with the anterior margin, furnished with rather irregular rows of minute brown hooks, the third, fourth, and fifth having a transverse fold, armed in the same way;† anus truncated, retracted, and wrinkled. Head subglobose, furnished with an elongated orifice (Fig. 3, *e*,) on each side behind; mouth with two tubercles terminated by a vesicle, or aperture (*c*), another above them (*d*), and two strong, horny, and black spines below (*b*).‡

It must be observed, that this description would not completely represent the living animal, on account of the parts being contracted and hardened by saturation in spirits of wine. This may account for my not having detected any spiracula, which are strongly developed, I believe, in the Œstri, but not in the Gasterophili. There were some hairs floating in the bottle, which, I think, never were attached to the larva.

It is well known that the female fly of the Œstrus bovis deposits her eggs on the back of the ox and cow, where they hatch, and, eating through the skin, form a tumour beneath: the same accident might therefore readily happen to a man, if his skin were exposed, during the heat of the day, where the flies abound; and the same consequences would follow.

It would be extremely desirable to ascertain the fly that the larva produces, and to accomplish this it may be useful to observe, that, if at any time a full-grown one should be obtained, a garden-pot, or other earthen vessel, nearly filled with rather moist earth, put in lightly, should be taken, and, the grub being placed on the top, it would soon bury itself, and change to a pupa, or chrysalis. If the pot were set in a saucer, and a little water poured into the latter, so that it

* Pallas mentions an Œstrus Hominis, but, whether it be the same as our insect, I have no means of ascertaining at present.

† These hooks probably serve two purposes: by irritation, they cause an additional secretion in the cavity where they live, and by them they are enabled to turn round and change their attitude, as well as to make their escape from the tumour, when full-grown. They seem to be formed like the thorns of a rose or brier, and most of them point towards the tail; but some, especially approaching the posterior part, are directed towards the head.

‡ These spines are supposed not to exist in the subcutaneous feeding larvæ that have been examined, they being provided for attaching the animal to the stomach, &c., as in the Œst. equi; but those of the sheep, living in the frontal sinuses, as well as of the deer, as already mentioned, are furnished with them.

might be gradually absorbed through the bottom of the garden-pot, it would be kept moist; a very essential state for producing the fly; and, to prevent its escape when it hatches,* a piece of gauze, or anything that is transparent, must be laid over the top, and the fly ought not to be killed until some hours after it has come out from the pupa, that the wings and other parts may have time to dry, and assume their natural form and colour.

*Saville row; November*, 1833.

* This sometimes takes place in a fortnight after the larva enters the earth; at other times, I believe, two or three months elapse.

## Figures and Tables

**Figure f1:**
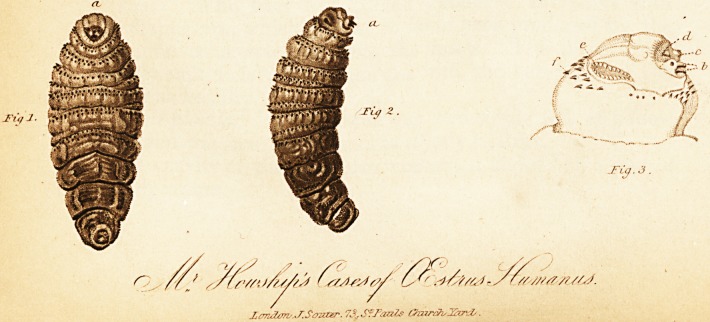


**Figure f2:**